# 2,3-Bis[(3-methyl­biphenyl-4-yl)imino]­butane

**DOI:** 10.1107/S1600536814005686

**Published:** 2014-03-19

**Authors:** Jingjing Chen, Jianchao Yuan, Jie Zhao, Weibing Xu, Yanqiong Mu

**Affiliations:** a Key Laboratory of Eco-Environment-Related Polymer Materials of the Ministry of Education, Key Laboratory of Polymer Materials of Gansu Province, College of Chemistry & Chemical Engineering, Northwest Normal University, Lanzhou 730070, People’s Republic of China

## Abstract

The title compound, C_30_H_28_N_2_, is a product of the condensation reaction of 2-methyl-4-phenyl­aniline and butane-2,3-dione. The mol­ecule lies on a crystallographic inversion centre. The C=N bond has an *E* conformation. The dihedral angle between the two benzene rings of the 4-phenyl-2-methyl­phenyl group is 29.19 (76)°. The 1,4-di­aza­butadiene plane makes an angle of 70.1 (10)° with the N-bonded methyl­phenyl ring and an angle of 81.08 (97)° with the terminal phenyl group.

## Related literature   

The title compound was synthesized as an α-di­imine ligand for applications in olefin polymerization Ni(II)-α-di­imine catalysts, see: Johnson *et al.* (1995 ▶); Killian *et al.* (1996 ▶); Wang *et al.* (2013 ▶); Ionkin & Marshall (2004 ▶); Meinhard *et al.* (2007 ▶). For the effect of the ligand structure on the activity of the catalyst and the properties of the products, see: Popeney & Guan (2005 ▶); Yuan *et al.* (2005 ▶); Helldörfer *et al.* (2003 ▶). For related structures, see: Yuan *et al.* (2013 ▶).
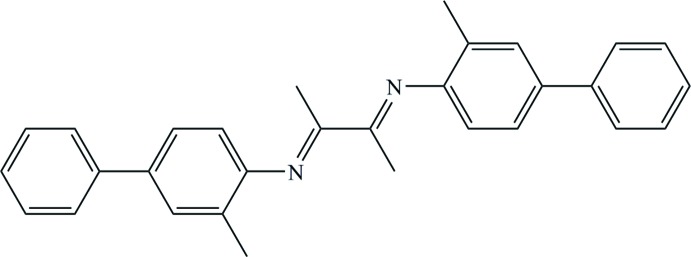


## Experimental   

### 

#### Crystal data  

C_30_H_28_N_2_
*M* *_r_* = 416.54
Orthorhombic, 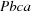

*a* = 8.347 (3) Å
*b* = 7.063 (3) Å
*c* = 39.946 (16) Å
*V* = 2355.0 (16) Å^3^
*Z* = 4
Mo *K*α radiation
μ = 0.07 mm^−1^
*T* = 293 K
0.19 × 0.18 × 0.15 mm

#### Data collection  

Bruker APEXII CCD diffractometer
Absorption correction: multi-scan (*SADABS*; Sheldrick, 2004 ▶) *T*_min_ = 0.987, *T*_max_ = 0.990
15668 measured reflections
2200 independent reflections
1251 reflections with *I* > 2σ(*I*)
*R*_int_ = 0.088

#### Refinement  

*R*[*F* ^2^ > 2σ(*F* ^2^)] = 0.069
*wR*(*F* ^2^) = 0.135
*S* = 1.01
2200 reflections
148 parameters
H-atom parameters constrained
Δρ_max_ = 0.15 e Å^−3^
Δρ_min_ = −0.16 e Å^−3^

### 

Data collection: *APEX2* (Bruker, 2008 ▶); cell refinement: *SAINT* (Bruker, 2008 ▶); data reduction: *SAINT*; program(s) used to solve structure: *SHELXTL* (Sheldrick, 2008 ▶); program(s) used to refine structure: *SHELXTL*; molecular graphics: *SHELXTL*; software used to prepare material for publication: *SHELXTL*.

## Supplementary Material

Crystal structure: contains datablock(s) I, New_Global_Publ_Block. DOI: 10.1107/S1600536814005686/fk2080sup1.cif

Structure factors: contains datablock(s) I. DOI: 10.1107/S1600536814005686/fk2080Isup2.hkl

Supporting information file. DOI: 10.1107/S1600536814005686/fk2080Isup3.cml

CCDC reference: 991458

Additional supporting information:  crystallographic information; 3D view; checkCIF report

## Figures and Tables

**Fig. 1.**
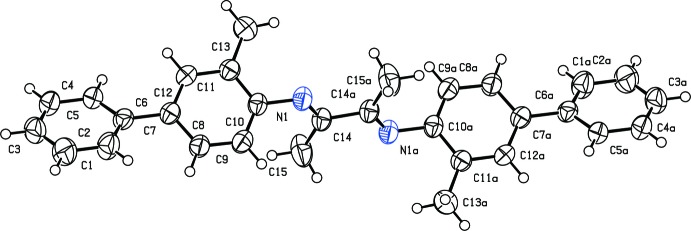
The title molecule with displacement ellipsoids plotted at 50% probability level. Atoms with label "a" are related by the symmetry code (-x+1, -y+1, -z).

**Fig. 2.**
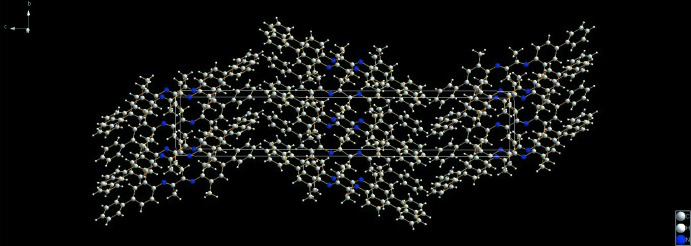
Crystal packing viewed along the a-axis.

## supplementary crystallographic information

### 1. Comment

In recent years, a variety of α-diimine ligands containing various *ortho* and *para* position substituted *N*-aryl rings (Johnson *et al.* 1995; Killian *et al.*. 1996; Popeney *et al.*. 2005; Yuan *et al.*, 2005; Wang *et al.* 2013) and backbone effects (Helldörfer *et al.*. 2003) and teraryl substituted-α-diimine ligands (Ionkin *et al.*. 2004; Meinhard *et al.*. 2007) were employed to study their influence on the catalytic activity of α-diimine-Ni(II) complexes. In this study, we designed and synthesized the title compound as a bidentate ligand. The molecule lies on a crystallographic inversion centre. The single bond of 1, 4-diazabutadiene fragment is (E)-configured. The dihedral angles between the 1,4-diazabutadiene plane and the benzene ring bonded to the N atom are 70.12 (96)° and 81.08 (97)° for the terminal phenyl group, resp. The dihedral angle between both aromatic ring planes is 29.19 (76)° (Figure 1). The crystal packing shows stacking of molecules along a-axis (Figure 2), however, no significant intermolecular H-bonding is observed. A very similar molecular structure is known from Yuan *et al.* (2013).

### 2. Experimental

Formic acid (0.5 ml) was added to a stirred solution of 2-methyl-4-phenylaniline (0.916 g, 2.2 mmol) and 2,3-Butanedione (0.086 g, 1 mmol) in 20 ml anhydrous ethanol (20 ml). The mixture was stirred at 50 *^o^*C for 24 h, then cooled, and the precipitate was separated by filtration. The solid was recrystallized from ethanol/dichloromethane (*v*/*v*= 8:1), washed with cold ethanol and dried under vacuum to give the title compound. Yield is 86%. Crystals suitable for X-ray structure determination were grown from a cyclohexane/dichloromethane (v:v= 1:2) solution. Anal. Calc. for C_30_H_28_N_2_: C, 86.50; H, 6.78; N, 6.72. Found: C, 86.62; H, 6.57; N, 6.58.

### 3. Refinement

Positions of the methyl H atoms were derived from Fourier maps (HFIX 137), with C–H 0.96 Å and *U*_iso_(H) = 1.5U_eq_(C). All other H atoms were placed in geometrically idealized positions and constrained to ride on their parent atoms with C–H distances distances of 0.93 Å and *U*_iso_(H) = 1.2U_eq_(C).

### Crystal data

**Table:**

C_30_H_28_N_2_	*D*_x_ = 1.175 Mg m^−^^3^
*M**_r_* = 416.54	Mo *K*α radiation, λ = 0.71073 Å
Orthorhombic, *P**b**c**a*	Cell parameters from 1674 reflections
*a* = 8.347 (3) Å	θ = 2.6–21.7°
*b* = 7.063 (3) Å	µ = 0.07 mm^−^^1^
*c* = 39.946 (16) Å	*T* = 293 K
*V* = 2355.0 (16) Å^3^	Block, yellow
*Z* = 4	0.19 × 0.18 × 0.15 mm
*F*(000) = 888	

### Data collection

**Table:**

Bruker APEXII CCD diffractometer	2200 independent reflections
Radiation source: fine-focus sealed tube	1251 reflections with *I* > 2σ(*I*)
Graphite monochromator	*R*_int_ = 0.088
φ and ω scans	θ_max_ = 25.5°, θ_min_ = 2.6°
Absorption correction: multi-scan (*SADABS*; Sheldrick, 2004)	*h* = −10→10
*T*_min_ = 0.987, *T*_max_ = 0.990	*k* = −8→8
15668 measured reflections	*l* = −48→47

### Refinement

**Table:**

Refinement on *F*^2^	Secondary atom site location: difference Fourier map
Least-squares matrix: full	Hydrogen site location: difference Fourier map
*R*[*F*^2^ > 2σ(*F*^2^)] = 0.069	H-atom parameters constrained
*wR*(*F*^2^) = 0.135	*w* = 1/[σ^2^(*F*_o_^2^) + (0.0366*P*)^2^ + 1.4273*P*] where *P* = (*F*_o_^2^ + 2*F*_c_^2^)/3
*S* = 1.01	(Δ/σ)_max_ < 0.001
2200 reflections	Δρ_max_ = 0.15 e Å^−^^3^
148 parameters	Δρ_min_ = −0.16 e Å^−^^3^
0 restraints	Extinction correction: *SHELXTL* (Sheldrick, 2008), Fc^*^=kFc[1+0.001xFc^2^λ^3^/sin(2θ)]^-1/4^
Primary atom site location: structure-invariant direct methods	Extinction coefficient: 0.0025 (6)

### Special details

**Table:**

Geometry. All e.s.d.'s (except the e.s.d. in the dihedral angle between two l.s. planes) are estimated using the full covariance matrix. The cell e.s.d.'s are taken into account individually in the estimation of e.s.d.'s in distances, angles and torsion angles; correlations between e.s.d.'s in cell parameters are only used when they are defined by crystal symmetry. An approximate (isotropic) treatment of cell e.s.d.'s is used for estimating e.s.d.'s involving l.s. planes.
Refinement. Refinement of *F*^2^ against ALL reflections. The weighted *R*-factor *wR* and goodness of fit *S* are based on *F*^2^, conventional *R*-factors *R* are based on *F*, with *F* set to zero for negative *F*^2^. The threshold expression of *F*^2^ > σ(*F*^2^) is used only for calculating *R*-factors(gt) *etc*. and is not relevant to the choice of reflections for refinement. *R*-factors based on *F*^2^ are statistically about twice as large as those based on *F*, and *R*- factors based on ALL data will be even larger.

### Fractional atomic coordinates and isotropic or equivalent isotropic displacement parameters (Å^2^)

**Table:**

	*x*	*y*	*z*	*U*_iso_*/*U*_eq_	
C1	0.6218 (4)	−0.0240 (5)	0.17712 (7)	0.0644 (9)	
H1	0.7117	−0.0468	0.1640	0.077*	
C2	0.6029 (4)	−0.1219 (5)	0.20680 (8)	0.0716 (10)	
H2	0.6792	−0.2106	0.2133	0.086*	
C3	0.4725 (4)	−0.0893 (5)	0.22677 (7)	0.0663 (9)	
H3	0.4597	−0.1548	0.2468	0.080*	
C4	0.3612 (4)	0.0413 (4)	0.21680 (7)	0.0594 (9)	
H4	0.2726	0.0652	0.2303	0.071*	
C5	0.3788 (4)	0.1375 (4)	0.18713 (6)	0.0504 (8)	
H5	0.3012	0.2248	0.1807	0.061*	
C6	0.5099 (3)	0.1070 (4)	0.16654 (6)	0.0442 (7)	
C7	0.5276 (3)	0.2107 (4)	0.13451 (6)	0.0425 (7)	
C8	0.6072 (3)	0.1298 (4)	0.10744 (7)	0.0509 (8)	
H8	0.6563	0.0124	0.1099	0.061*	
C9	0.6142 (3)	0.2216 (4)	0.07704 (7)	0.0532 (8)	
H9	0.6678	0.1652	0.0592	0.064*	
C10	0.5427 (3)	0.3961 (4)	0.07266 (6)	0.0423 (7)	
C11	0.4688 (3)	0.4856 (4)	0.09956 (6)	0.0432 (7)	
C12	0.4629 (3)	0.3887 (4)	0.12980 (6)	0.0443 (7)	
H12	0.4126	0.4468	0.1479	0.053*	
C13	0.3949 (4)	0.6774 (4)	0.09594 (8)	0.0674 (10)	
H13A	0.2882	0.6648	0.0873	0.101*	
H13B	0.4580	0.7522	0.0808	0.101*	
H13C	0.3912	0.7384	0.1174	0.101*	
C14	0.4850 (3)	0.4425 (4)	0.01552 (6)	0.0440 (7)	
C15	0.3729 (4)	0.2792 (5)	0.01278 (8)	0.0773 (11)	
H15A	0.3722	0.2102	0.0335	0.116*	
H15B	0.4073	0.1972	−0.0050	0.116*	
H15C	0.2670	0.3248	0.0081	0.116*	
N1	0.5572 (3)	0.4954 (3)	0.04174 (5)	0.0486 (6)	

### Atomic displacement parameters (Å^2^)

**Table:**

	*U*^11^	*U*^22^	*U*^33^	*U*^12^	*U*^13^	*U*^23^
C1	0.068 (2)	0.078 (3)	0.0472 (19)	0.013 (2)	0.0012 (16)	0.0083 (18)
C2	0.086 (3)	0.075 (2)	0.054 (2)	0.016 (2)	−0.0096 (19)	0.012 (2)
C3	0.092 (3)	0.064 (2)	0.0429 (18)	−0.005 (2)	−0.0080 (19)	0.0083 (17)
C4	0.075 (2)	0.063 (2)	0.0400 (17)	−0.0046 (19)	0.0066 (16)	−0.0005 (16)
C5	0.0651 (19)	0.0476 (19)	0.0386 (17)	−0.0013 (16)	0.0034 (14)	0.0006 (15)
C6	0.0502 (17)	0.0475 (18)	0.0349 (15)	0.0018 (15)	−0.0037 (13)	−0.0025 (14)
C7	0.0388 (15)	0.0504 (19)	0.0381 (16)	−0.0011 (15)	−0.0014 (13)	−0.0022 (14)
C8	0.0587 (18)	0.0529 (19)	0.0410 (17)	0.0119 (16)	0.0057 (14)	0.0047 (16)
C9	0.0593 (18)	0.058 (2)	0.0424 (18)	0.0049 (17)	0.0088 (15)	−0.0019 (16)
C10	0.0417 (15)	0.0495 (18)	0.0356 (16)	−0.0052 (15)	−0.0010 (13)	0.0013 (14)
C11	0.0430 (15)	0.0456 (17)	0.0410 (17)	−0.0007 (14)	−0.0023 (13)	−0.0011 (14)
C12	0.0474 (16)	0.0479 (18)	0.0377 (16)	0.0014 (15)	0.0034 (13)	−0.0058 (14)
C13	0.088 (2)	0.055 (2)	0.059 (2)	0.0099 (19)	0.0034 (18)	0.0063 (17)
C14	0.0404 (15)	0.0522 (19)	0.0395 (16)	−0.0047 (14)	0.0022 (13)	0.0024 (13)
C15	0.090 (2)	0.086 (3)	0.055 (2)	−0.041 (2)	−0.0109 (19)	0.0185 (19)
N1	0.0521 (14)	0.0561 (16)	0.0375 (13)	−0.0075 (12)	0.0012 (12)	0.0028 (12)

### Geometric parameters (Å, º)

**Table:**

C1—C6	1.381 (4)	C9—C10	1.380 (4)
C1—C2	1.382 (4)	C9—H9	0.9300
C1—H1	0.9300	C10—C11	1.391 (3)
C2—C3	1.369 (4)	C10—N1	1.426 (3)
C2—H2	0.9300	C11—C12	1.389 (3)
C3—C4	1.368 (4)	C11—C13	1.496 (4)
C3—H3	0.9300	C12—H12	0.9300
C4—C5	1.374 (4)	C13—H13A	0.9600
C4—H4	0.9300	C13—H13B	0.9600
C5—C6	1.386 (4)	C13—H13C	0.9600
C5—H5	0.9300	C14—N1	1.265 (3)
C6—C7	1.481 (4)	C14—C15	1.489 (4)
C7—C12	1.381 (4)	C14—C14^i^	1.504 (5)
C7—C8	1.392 (3)	C15—H15A	0.9600
C8—C9	1.378 (4)	C15—H15B	0.9600
C8—H8	0.9300	C15—H15C	0.9600
			
C6—C1—C2	121.4 (3)	C8—C9—H9	119.6
C6—C1—H1	119.3	C9—C10—C11	120.0 (3)
C2—C1—H1	119.3	C9—C10—N1	120.8 (2)
C3—C2—C1	120.4 (3)	C11—C10—N1	118.9 (3)
C3—C2—H2	119.8	C10—C11—C12	117.6 (3)
C1—C2—H2	119.8	C10—C11—C13	121.3 (3)
C4—C3—C2	118.9 (3)	C12—C11—C13	121.1 (3)
C4—C3—H3	120.5	C7—C12—C11	123.6 (3)
C2—C3—H3	120.5	C7—C12—H12	118.2
C3—C4—C5	120.8 (3)	C11—C12—H12	118.2
C3—C4—H4	119.6	C11—C13—H13A	109.5
C5—C4—H4	119.6	C11—C13—H13B	109.5
C4—C5—C6	121.3 (3)	H13A—C13—H13B	109.5
C4—C5—H5	119.4	C11—C13—H13C	109.5
C6—C5—H5	119.4	H13A—C13—H13C	109.5
C1—C6—C5	117.2 (3)	H13B—C13—H13C	109.5
C1—C6—C7	121.9 (3)	N1—C14—C15	126.1 (3)
C5—C6—C7	120.9 (3)	N1—C14—C14^i^	116.4 (3)
C12—C7—C8	117.0 (3)	C15—C14—C14^i^	117.5 (3)
C12—C7—C6	121.9 (3)	C14—C15—H15A	109.5
C8—C7—C6	121.1 (3)	C14—C15—H15B	109.5
C9—C8—C7	120.8 (3)	H15A—C15—H15B	109.5
C9—C8—H8	119.6	C14—C15—H15C	109.5
C7—C8—H8	119.6	H15A—C15—H15C	109.5
C10—C9—C8	120.9 (3)	H15B—C15—H15C	109.5
C10—C9—H9	119.6	C14—N1—C10	122.1 (2)
			
C6—C1—C2—C3	−0.8 (5)	C8—C9—C10—C11	3.0 (4)
C1—C2—C3—C4	0.2 (5)	C8—C9—C10—N1	176.7 (3)
C2—C3—C4—C5	0.5 (5)	C9—C10—C11—C12	−3.4 (4)
C3—C4—C5—C6	−0.6 (5)	N1—C10—C11—C12	−177.2 (2)
C2—C1—C6—C5	0.7 (5)	C9—C10—C11—C13	178.0 (3)
C2—C1—C6—C7	−179.0 (3)	N1—C10—C11—C13	4.2 (4)
C4—C5—C6—C1	0.0 (4)	C8—C7—C12—C11	2.3 (4)
C4—C5—C6—C7	179.7 (3)	C6—C7—C12—C11	−176.1 (3)
C1—C6—C7—C12	−152.1 (3)	C10—C11—C12—C7	0.7 (4)
C5—C6—C7—C12	28.3 (4)	C13—C11—C12—C7	179.4 (3)
C1—C6—C7—C8	29.6 (4)	C15—C14—N1—C10	2.3 (5)
C5—C6—C7—C8	−150.0 (3)	C14^i^—C14—N1—C10	−178.4 (3)
C12—C7—C8—C9	−2.7 (4)	C9—C10—N1—C14	71.9 (4)
C6—C7—C8—C9	175.7 (3)	C11—C10—N1—C14	−114.4 (3)
C7—C8—C9—C10	0.1 (4)		

Symmetry code: (i) −*x*+1, −*y*+1, −*z*.
